# Simvastatin treatment varies the radiation response of human breast cells in 2D or 3D culture

**DOI:** 10.1007/s10637-020-01046-6

**Published:** 2020-12-11

**Authors:** Katrin Manda, Dajana Juerß, Paul Fischer, Annemarie Schröder, Annelie Koenen, Guido Hildebrandt

**Affiliations:** Department of Radiotherapy and Radiation Oncology, University Medical Center Rostock, Suedring 75, 18059 Rostock, Germany

**Keywords:** MCF-7, MCF10A, Simvastatin, Radiation, 3D

## Abstract

*Background* Statins inhibit the cholesterol biosynthesis and are used as cholesterol-lowering agents in fat-metabolism disorders. Furthermore, several studies state that statins have supportive functions in breast cancer treatment. Therefore, simvastatin (SVA) as a potential radiosensitizer should be investigated on the basis of human breast cells. *Methods* First, an optimal concentration of SVA for normal (MCF10A) and cancer (MCF-7) cells was identified via growth and cytotoxicity assays that, according to the definition of a radiosensitizer in the narrower sense, enhances the effect of radiation therapy but has no cytotoxic effect. Next, in combination with radiation SVA’s influence on DNA repair capacity and clonogenic survival in 2D and 3D was determined. Furthermore cell cycle distribution, expression of survivin and connective tissue growth factor (CTGF) as well as ERK1 map kinase were analysed. *Results* 1 μM SVA was identified as highest concentration without an influence on cell growth and cytotoxicity and was used for further analyses. In terms of early and residual γH2AX-foci, SVA affected the number of foci in both cell lines with or without irradiation. Different radiation responses were detected in 2D and 3D culture conditions. During the 2D cultivation, a radiosensitizing effect within the clonogenic survival was observable, but not in 3D. *Conclusion* The present study suggests that SVA may have potential for radiosensitization. Therefore, it is important to further investigate the role of SVA in relation to the extent of radiosensitization and how it could be used to positively influence the therapy of breast cancer or other entities.

**Electronic supplementary material:**

The online version of this article (10.1007/s10637-020-01046-6) contains supplementary material, which is available to authorized users.

## Introduction

Simvastatin (SVA) belongs to the group of statins which act as competitive inhibitors of the 3-hydroxy-3-methylglutaryl coenzyme A (HMG-CoA)-reductase and are used as cholesterol-lowering drugs. In this function, statins directly inhibit the formation of mevalonate from HMG-CoA and thus also the biosynthesis of cholesterol [1].

But next to its cholesterol-lowering effect, several studies mention statins’ preventive and supportive functions before, during, and after cancer treatment [2–5]. In breast cancer patients for example, decreased recurrence rates relating to the use of statins were described [6, 7]. Beckwitt et al. (2018) have summarized the potential of statins to reduce the progression and mortality of breast cancer; they have supported the use of statins as a secondary prevention measure [8]. Within the scope of preoperative treatment with statins, a reduced tumour cell proliferation rate in breast cancer patient samples, measured by the KI67-expression, was observed [9]. Owing to its beneficial efforts for breast cancer patients, it was supported as a component of adjuvant therapy [10]. But, in fact, not all tumour entities are equally sensitive to statins, there are also studies that found no beneficial effect of them in a colon cancer cohort [11] and for triple negative breast cancer patients [12].

While exploring the beneficial effort of statins, experimental studies have shown a sensitizing effect on tumour cells, either in combination with chemotherapeutics [13, 14] or with radiation treatment [15, 16]. A recently published study reveals that a combined treatment of SVA and a Vitamin E analogue led to an enhancement of radiation protection in mice [17]. Two other experimental studies have already proven the radioprotective effect of a further statin, lovastatin. First, it was observed in vivo that a reduced pro-inflammatory radiation response after lovastatin treatment caused a decreased level of radiation-induced normal tissue damage [18]. Second, incubation with lovastatin resulted in creating some kind of protection for endothelial cells [19] and combined with doxorubicin of keratinocytes [20] against the ionizing radiation (IR)-induced cell death.

But on what mechanisms is the supportive effect of statins based? It have been shown that SVA alone or SVA combined with a Vitamin E analogue supports the induction of an anticoagulant with radio-protective efficacy [17]. Another supportive effect could be that SVA can elicit an inhibition of the ATP-binding cassette (ABC) transporters like ABCB1 (P-glycoprotein), which can promote drug-resistance by repressing chemotherapeutics so that drug treatment on different tumour cell lines could be improved through application of SVA. [21]. It could be assumed that as basic mechanisms, for example, posttranslational and epigenetic modifications would ensure the effect of statins. A downregulation of the expression of DNMT1, a key player in epigenetic regulation, and further epigenetic changes, such as downregulation of histone deacetylases, on different tumour cells caused by SVA treatment have been detected [22].

Until now, there has been less experimental data on the effect of statins on normal human cells of the breast. While in epidemiological studies statins showed a beneficial effort for patients, as described above, it could be assumed that the resultant radiation protection is one mechanism of the positive effect of statins.

Owing to the large use of SVA, the present study focuses on the investigation of the potentially radioprotective effect of the drug on normal cells and the possible radiosensitizing effect on cancer cells, both in the human breast. Additionally, it has been tested whether the effect of SVA on the cellular radiation response differs between 2D and 3D cell-culturing conditions. In 3D cell culture conditions, cells behave more similar to the in vivo situation than in the 2D cell culture conditions. Moreover, it is known that the radiation response differs between 2D and 3D cell cultures [23].

To verify the potential of SVA as a possible supporting drug with less side effects in radiotherapy, our study investigates more closely the influence of SVA on cancer and normal cells of the human breast and their radiation responses.

## Methods

### Cell culture and simvastatin treatment

In the present study, MCF10A (provided by Prof. Kevin Prise, Queen’s University Belfast, Ireland*)*, a spontaneously transformed cell line from normal human breast epithelial cells [24], and MCF-7 (ATCC; HTB-22™), a breast cancer cell line, were used. The MCF10A cells were cultivated using Dulbecco’s modified Eagle medium/F12 (DMEM/F12, Gibco/Life Technologies, Darmstadt, Germany) supplemented with 0.01% cholera toxin, 0.1% insulin, 0.05% hydrocortisone and 1% penicillin/streptomycin (all Sigma Aldrich, Hamburg, Germany), 0.02% epidermal growth factor (EGF; Gibco/Life Technologies), and 5% horse serum (Fisher Scientific, Schwerte, Germany). The MCF-7 and MDA-MB-231 cells were cultured in DMEM media (Lonza/Biozym, Basel, Switzerland) supplemented with 10% foetal calf serum (Biochrom GmbH, Berlin, Germany) and 1% penicillin/streptomycin (Sigma-Aldrich Chemie GmbH, Taufkirchen, Germany). All three cell lines were cultivated under 5% CO_2_ at 37 °C and passaged two times a week using a 0.05% trypsin/EDTA solution (Biochrom GmbH, Berlin, Germany).

Simvastatin (SVA; Sigma Aldrich) was solved in DMSO (Merck, Darmstadt, Germany) and added to the cells in different concentrations (0.05 μM, 0.1 μM, 0.5 μM, 1 μM, 3 μM). A control for DMSO, as a solvent, was also carried out relating to the DMSO level in the highest level of SVA application. SVA was always added 24 h after seeding and before irradiation treatment.

### Irradiation

Using the Linac Siemens Oncor Expression (Healthcare Sector Siemens AG, Erlangen, Germany), the cells were irradiated 48 h after seeding at a dose rate of 3.75 Gy/min. The irradiation doses used were 0.5 Gy, 2 Gy, 4 Gy, and 6 Gy. Sham irradiated samples were used as negative control.

### Growth curves

1 × 10^4^ cells were seeded as triplicates in multi-well plates for each SVA value. After 24 h, SVA was added in different concentrations. By using 0.25% trypsin/EDTA solution (Biochrom GmbH), the cell number of three untreated wells was determined at the same time point. Up to six days after the SVA application, the cell number for each SVA value and control was detected.

### Measurement of cytotoxicity

Two days before IR treatment, the MCF10A (2 × 10^3^) and MCF-7 (7.5 × 10^3^) cells of both cell lines were seeded in multi-well plates as triplicates for each dose value. Subsequently, SVA was added 24 h later. Using the Pierce LDH Cytotoxicity Assay Kit (Thermo Fisher Scientific, Darmstadt, Germany), the cytotoxicity was detected for each value. The LDH Cytotoxicity Assay Kit was performed in keeping with the manufacturer’s instructions. Based on the principle that the accessible enzyme lactate dehydrogenase can metabolize the tetrazolium salt into formazan only in dead cells, the metabolized formazan dye quantity is directly correlated with the number of dead cells. The measurement was performed with an ANTHOS zenyth 340r reader (Anthos Mikrosysteme GmbH, Krefeld, Germany).

### Immunostaining of DSBs via γH2AX antibody

Two days before IR treatment, 5 × 10^4^ (MCF-7) or 2.5 × 10^4^ (MCF10A) cells per well (1.8 cm^2^) were seeded as duplicates in chamber slides (LabTek®, Nunc, Roskilde, Denmark). The addition of SVA was performed 24 h later. After fixation with 2% formaldehyde and permeabilization with 0.25% triton-X 100 (both Sigma Aldrich Chemie GmbH, Munich, Germany), the cells were consecutively incubated for 60 min with the anti-γH2AX antibody (1:500, clone JBW301, Merck Millipore) and Alexa Fluor 594 goat anti-mouse IgG1 (1:400, Molecular Probes®/Life Technologies, Darmstadt, Germany) for 30 min. The slides were mounted with Vectashield® containing anti-4′,6-diamidino-2-phenylindole (DAPI; Vector Laboratories, Inc., Burlingame, CA). The foci were visualized with an Eclipse TE300 inverted microscope (Nikon, Tokyo, Japan). At the magnification of 1000x, the foci of 50 cells per chamber were counted; two chambers per SVA concentration and irradiation dose were analysed.

### Colony-forming assay in 2D culture

Two days before the IR treatment, 1 × 10^3^ cells of MCF10A, MCF7 and MDA-MB-231 cell lines were seeded in 25 cm^2^ cell culture flasks as duplicates for each dose value. Subsequently, SVA was added after 24 h. A medium exchange was performed two days (MCF10A) or five days (MCF-7, MDA-MB-231) after the IR treatment. Eight days (MCF10A), 10 days (MDA-MB-231) or 14 days (MCF-7) after the seeding, the colonies were fixed with 70% ethanol for 10 min and stained for 5–10 min with 1% crystal violet solution (Serva Electrophoresis GmbH, Heidelberg, Germany). Colonies comprising 50 cells and more were counted. Afterwards, the survival fraction (SF) was determined.

### Colony-forming assay in 3D culture

For the colony-forming assay (CFA) performed in three-dimensional (3D) cultures [25], the multi-well plates were pre-coated with agarose (Thermo Fisher Scientific GmbH, Dreieich, Germany) in a final concentration of 1%. Afterwards, the 1 × 10^3^ cells, embedded in growth factor-reduced Matrigel™ (Corning Incorporated, NY, USA) with a final protein concentration of 0.5 mg/ml, were plated on the agarose layer 48 h before irradiation. Analogous to the CFA in 2D, SVA was added 24 h before the IR treatment. CFA in 3D were cultured under standard conditions. A medium exchange was performed once a week. For SF determination, the unfixed and unstained colonies were counted after 14 days.

### Cell cycle analysis

Cells were seeded in an appropriate density followed by medium exchange with serum free medium 24 h after cell seeding to induce synchronisation of cell cycle. Further 24 h later, SVA was added in different concentrations (0.05 μM, 0.1 μM, 0.5 μM, 1 μM). The irradiation with single-doses of 2 Gy or 0 Gy (control) for each experimental approach was performed 24 h after SVA addition and carried out at least in three independent experiments. 24 h or 72 h after irradiation cells were fixed and permeabilized 10 min in ethanol (70% (*v*/v), −20 °C), and stained with propidium iodide (75 μM). Samples were measured on flow cytometer Cytomics FC 500 (Beckman Coulter, Krefeld, Germany). Analysis was performed using Multicycle for Windows, version 3.0 (Phoenix Flow Systems, San Diego, USA).

### Measurement of marker survivin, CTGF and ERK1

To determine the release of survivin, human connective tissue growth factor (CTGF) and extracellular signal-regulated kinase 1 (ERK1) enzyme-linked immunosorbent assays (ELISA) were used. 1 × 10^4^ MCF10A or 1 × 10^4^ MCF-7 cells were seeded into each well of a 12-well plate. 1 μM of SVA was added 24 h later. At the end point of 48 h after irradiation, culture medium samples were collected, centrifuged, shock-frozen by means of nitrogen and stored at −80 °C until assayed. Simultaneously, the cells were detached by 0.05% trypsin/EDTA solution and centrifuged at 250 x g. Cell pellets were washed twice with PBS and lysed at 1 × 10^7^ cells/ml Lysis Buffer (R & D Systems, USA) on ice. The concentration of survivin and CTGF in the cell culture supernatant was assayed using the Human Survivin Quantikine ELISA Kit and the Human/CCN2 DuoSet ELISA (R & D Systems). The content of ERK1 in cell lysates was determined using the Human Total ERK1 DuoSet IC ELISA (R&D Systems). Optical densities were read using Anthos Zenyth 340 Plate Reader. The respective standard curves and protein concentrations were calculated via “Four Parameter Logistic Curve” online data analysis tool, MyAssays Ltd., 24 th. November 2012, http://www.myassays.com/four-parameter-logistic-curve.assay.

### Statistical analysis

Data of at least three independent experiments is represented in all figures as mean values ± standard deviation (SD) or standard error of mean (SEM). A value of *p* < 0.05 was considered to indicate a statistically significant difference. For comparing the sphere numbers, the statistical significance to the unirradiated control of each dimension (2D and 3D) was calculated via the one-sample t-test and a value of *p* < 0.02 indicated a statistically significant difference.

## Results

### Investigation of cell growth and potential cytotoxicity after SVA treatment

**Fig. 1:**
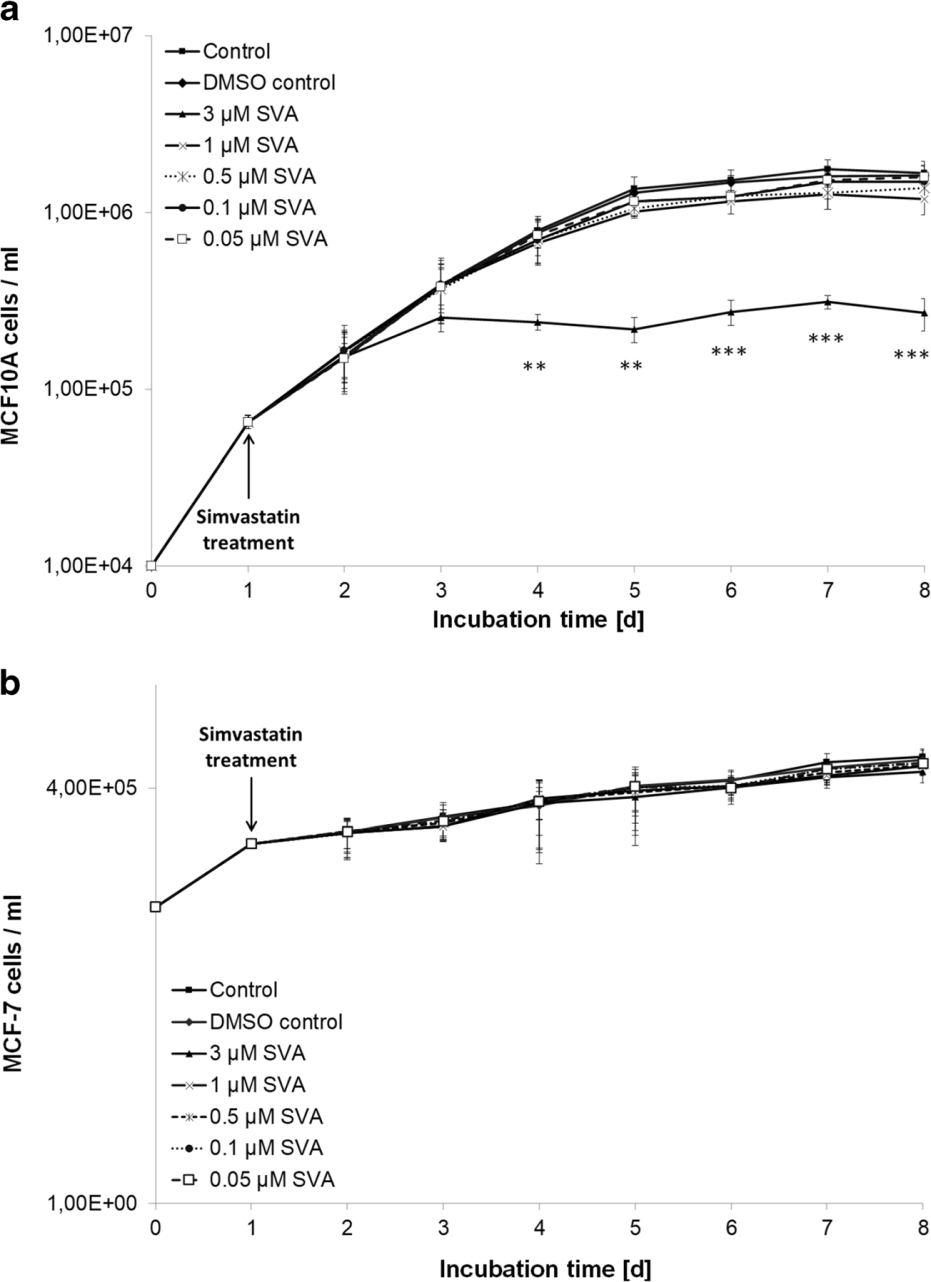
**Growth curves of normal cells MCF10A (a) and tumour cells MCF-7 (b) under influence of simvastatin.** Simvastatin (SVA) was added in different doses 24 h after seeding. Every day triplicates were scored for both cell lines. Data from three independent experiments are presented as mean values ± SD. Asterisks illustrate significances: ** *p* < 0.01, *** *p* < 0.001

Only compounds are defined as radiosensitizer in a narrower sense, which increase the effect of radiotherapy but do not have a cytotoxic effect even in the administered concentration [26]. Therefore the first step was to find a concentration of SVA that is non-toxic to both cell lines and that could then be used for all further experiments. The MCF10A and MCF-7 cells were treated with different concentrations of SVA and cell growth was analysed (Fig. 1). In both cell lines, the cell number was affected with increasing SVA concentrations in a dose-dependent manner. The MCF10A normal cells were more sensitive than the MCF-7 tumour cells. The MCF10A cells treated with 3 μM showed a stagnation of the cell number during the experiment, which was significant from Day 3, whereas the MCF-7 cells treated with the same SVA concentration showed only slightly lower cell amounts compared to the untreated control cells. Additionally, the cytotoxicity of these different doses of SVA was determined by using the LDH assay. All the tested SVA concentrations showed no cytotoxicity on both cell lines (Supplement 1). As described above, a SVA concentration was needed which would not affect the MCF10A and MCF-7 cells on its own. By using cytotoxic analysis and growth curves, SVA concentrations up to 1 μM were identified for further analyses.

### Repair capacity and residual γH2AX foci induction

**Fig. 2:**
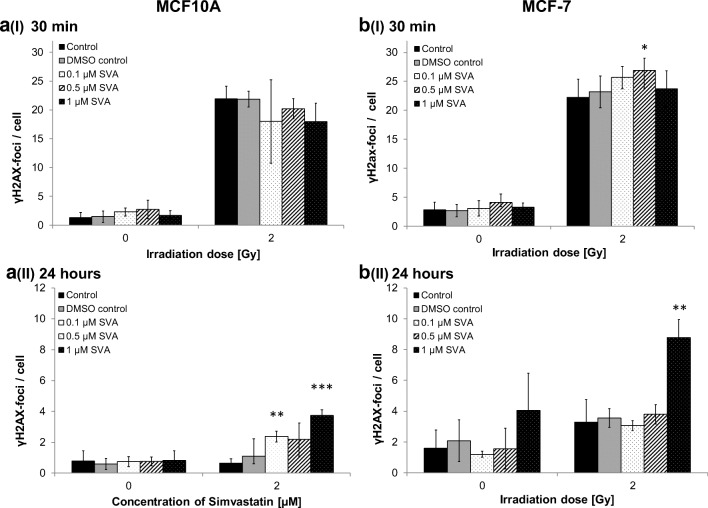
**DNA double-strand breaks under different simvastatin concentration after irradiation.** γH2AX foci were scored in MCF10A (**a**) and MCF-7 (**b**) cells 30 min (I) and 24 h (II) after radiation with 2 Gy and treatment with different doses of simvastatin (0.1 μM, 0.5 μM and 1 μM SVA). Data from three (MCF10A) and four (MCF-7) independent experiments are presented as mean values ± SD. Asterisks illustrate significances: * *p* < 0.05, ** p < 0.01, *** p < 0.001

The detection of γH2AX foci was performed to determine the number of DNA double-strand breaks for investigating the DNA repair capacity after treatment with SVA and irradiation. The γH2AX foci were analysed at an early time point, 30 min after the IR treatment and after a repair time of 24 h (Fig. 2).

Overall, it could be observed that the number of γH2AX foci was clearly higher in the MCF-7 cells than MCF10A. As expected, an irradiation of 2 Gy increased the number of γH2AX foci in both cell lines 30 min after the IR treatment. The influence of SVA within this time point was mostly not significant. Only a treatment with 0.5 μM on the MCF-7 cells showed significant changes. It also indicates that in comparison with control without the drug with increasing concentrations of SVA, the number of γH2AX foci decreased for MCF10A. But this effect was reversed for MCF-7, while the number of γH2AX foci increased with increasing SVA concentrations compared to control.

Also, 24 h after the irradiation, a different pattern of the γH2AX foci number within the two cell lines was observed. In the MCF10A cells, increasing SVA concentrations caused a continuous elevation in the mean number of DSBs for the irradiated cells, which was significant for 0.1 μM and 1 μM of SVA. Without irradiation, the number of γH2AX foci was not affected in the MCF10A cells. In the MCF-7 cells, a concentration of 1 μM of SVA showed an increase of DSBs—this was significant when the cells were additionally irradiated; lower doses of SVA caused no increase in the number of γH2AX foci. Interestingly, the unirradiated MCF-7 cells seemed sensitive for SVA; 0.1 μM and 0.5 μM elicited a decrease in the number of γH2AX foci, whereas 1 μM of SVA slightly increased the amount of γH2AX foci.

### Clonogenic survival of cells cultivated in 2D and 3D

**Fig. 3:**
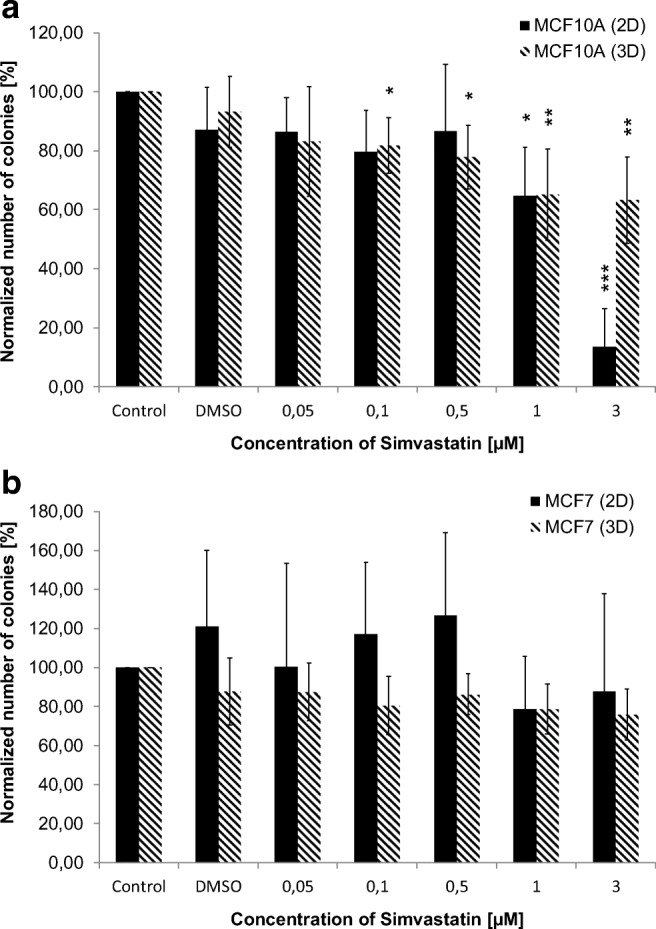
**Comparison of the effects of simvastatin (SVA) on colony formation in 2D and 3D without radiation.** SVA treatment was performed 24 h after seeding of MCF10A (**a**) and MCF-7 (**b**). The amount of colonies was determined two weeks after seeding. Data from at least three independent experiments are presented as normalized mean values of numbers of colonies ± SD. Statistical analysis was performed by using one-sided t-test. Asterisks illustrate significances: * *p* < 0.02, ** p < 0.01, *** *p* < 0.002

Using the clonogenic survival assay, long-term effects after the SVA treatment and radiation were investigated. In general, without irradiation, the number of colonies from the MCF10A cells decreased with increasing SVA concentrations compared to untreated control (Fig. 3). In 2D, significant changes were detected at 1 μM SVA and higher concentrations. In contrast, in 3D, the colony formation of the MCF10A cells was already affected in lower concentrations (0.1 μM up to 3 μM). For the MCF-7 cells, a significant influence of SVA on the number of colonies was not observable.

**Fig. 4:**
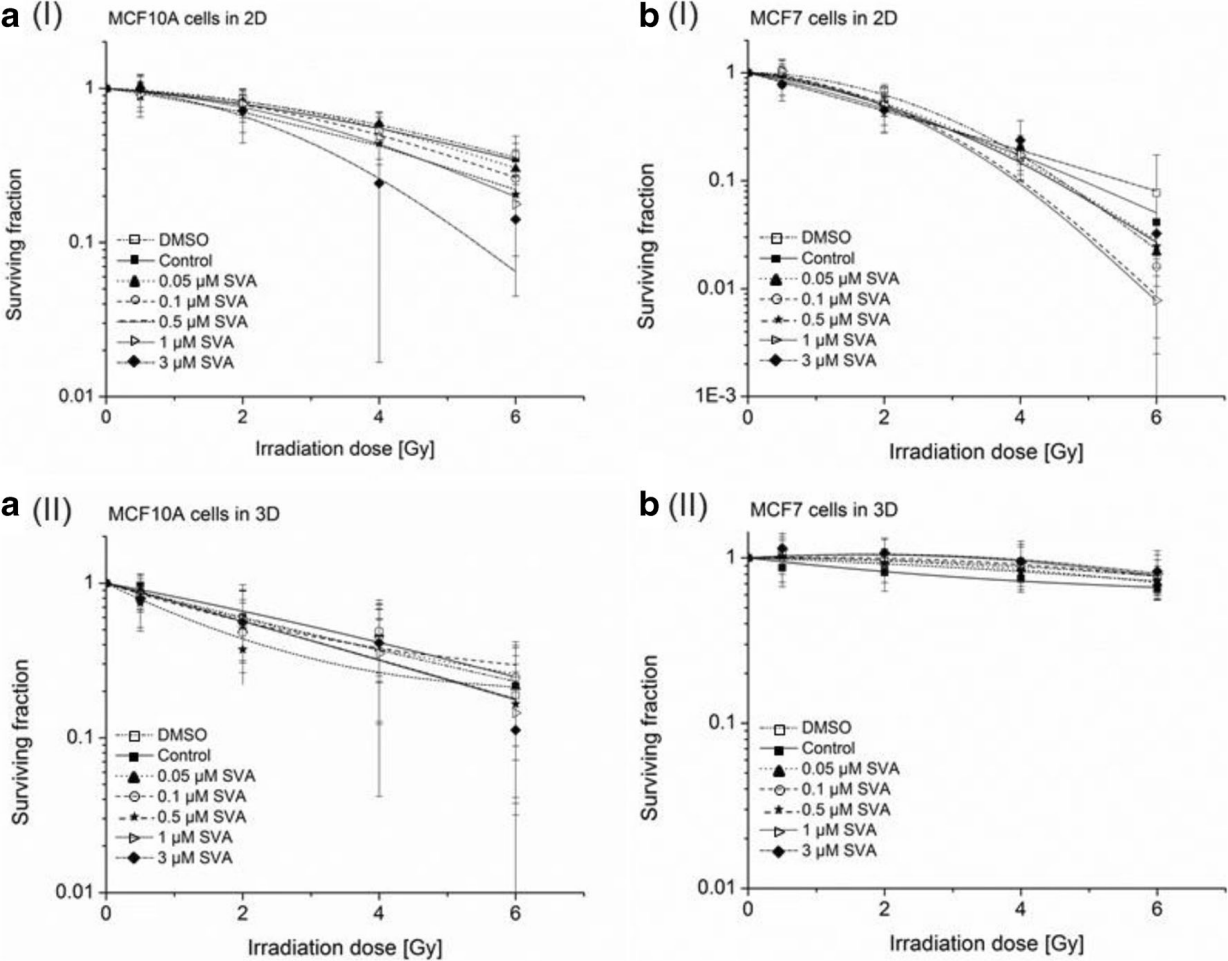
**Clonogenic survival after simvastatin (SVA) treatment of normal cells MCF10A (a) and tumour cells MCF-7 (b) in 2D (I) and 3D (II). **SVA treatment was performed 24 h after seeding and 24 h before irradiation treatment of cells. Data from at least three independent experiments are presented as normalized mean values of numbers of colonies ± SD. Statistical analysis was performed by Students t-test

After the irradiation treatment for all concentrations of SVA and the control (0 μM SVA), a dose-dependent decrease in the survival fraction (SF) could be observed (Fig. 4). However, for the MCF-7 cells, this effect was different between the cells cultivated in 2D and 3D. The MCF-7 cells were more radiosensitive when cultured in 2D with a survival fraction at 6 Gy (SF6) of 1.8 (0 μM SVA) and only 0.1 (1 μM SVA; Fig. 4b (I)). On the other hand, in the 3D cell culture, the SF6 of MCF-7 cells decreased to 6.4 (0 μM SVA) and 8.0 (1 μM SVA; Fig. 4b (II)). Such a different effect of the SF after 2D and 3D cell cultivations was not observed in the MCF10A cells. Cultivated in 2D, the SF6 of the MCF10A cells were 3.3 (0 μM SVA) and 1.5 (3 μM SVA), whereas in 3D the SF6 were 2.1 (0 μM SVA) and 1.1 (3 μM SVA; Fig. 4a (I) and A (II)). Next to this, in both 2D and 3D, no radioprotective effect of SVA could be observed on normal breast cells, namely MCF10A.

### Distribution of cell cycles

**Fig. 5:**
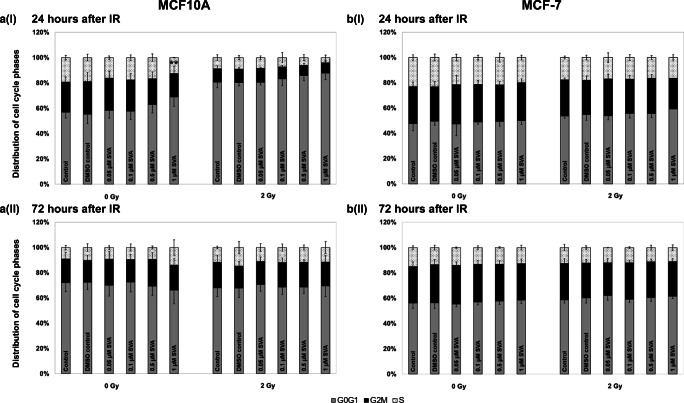
**Cell cycle analyses of simvastatin (SVA) treated MCF10A (a) and MCF-7 (b) cells in combination with ionizing radiation (2 Gy) or non-irradiation (0 Gy).** SVA was added to the cells 24 h before irradiation. Cells were fixed 24 h (I) or 72 h (II) after irradiation. For the three independent experiments significances were calculated in relation to non-irradiated controls without SVA treatment and illustrated by asterisks ** *p* ≤ 0.005

To investigate the cell cycle distribution of A) MCF10A and B) MCF-7 cells, flow cytometry was used to determine the ratio of cell cycle phases (Fig. 5). Generally, treatment with SVA did not influence the distribution of cells in the cell cycle phases of both cell lines, regardless of whether cells were non-irradiated or irradiated. Only after a high SVA concentration (1 μM) 48 h after treatment with the drug an increase of MCF10A cells in G0G1 phase and a significant decrease of cells in S phase could be observed. Additional irradiation had an clear effect on cell cycle distribution only in normal cells, resulting in an accumulation of cells being in G0/G1 phase. However, this effect was independently of SVA treatment.

Additionally, after treatment with SVA (± irradiation) in both cell lines no sub-G1 fraction as an indication of an apoptosis could be detected. The extension of incubation time to 72 h after irradiation showed similar effects on cell cycle distribution for cells.

### Expression of marker survivin, CTGF and ERK1

**Fig. 6:**
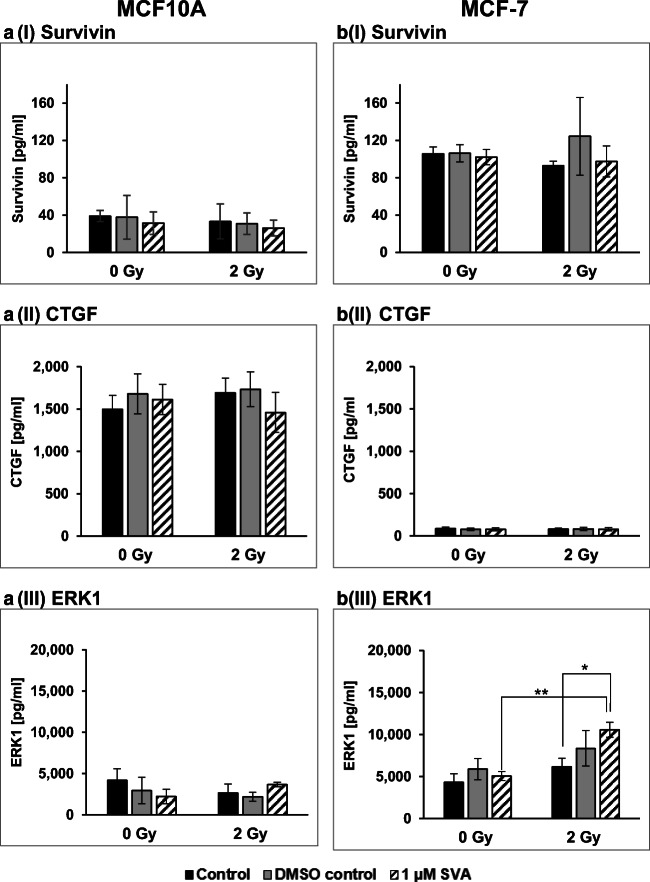
**Level of Survivin (I), Connective Tissue Growth Factor (CTGF, II) in supernatant and human extracellular signal-regulated kinase 1 (ERK1, III) in cell lysate of MCF10A (a) and MCF-7 (b) cells after therapy with simvastin (SVA) or/and irradiation**. The protein concentrations of these marker were determined by ELISA, 48 h after irradiation with 2 Gy. Results are illustrated as mean values ± SD of three independent experiments. Asterisks illustrate significant differences: * p < 0.05, ** *p* < 0.005. Statistical analyses were performed by two-tailed t-test

Since it is known that SVA treatment in combination with ionizing radiation influences the expression of survivin and connective tissue growth factor (CTGF) as well as extracellular-signal regulated kinase 1 (ERK1), all three markers in the breast cells were examined in more detail after treatment (Fig. 6). Except for ERK1 there was a clear difference in content between the normal MCF10A cells and the MCF-7 tumour cells for the markers. As was to be expected, the survivin level in the tumour cells was higher than in the normal cells; it was the other way round for CTGF levels. However, neither after treatment with SVA or radiation alone nor in a combination of both applications a change in the marker level of survivin and CTGF was detectable. For ERK1, the expression was approximately at the same level in both cell lines. Individual treatment with the drug did not show any change in the ERK1 level. However, the combination of both therapies led to an significant increase in the marker content, but only in the MCF-7 tumour cells.

## Discussion

To investigate the influence of SVA on the radiation response of cancer cells and normal tissue cells of the human breast, the present study examined different radiobiological effects following the SVA treatment in combination with IR. Currently, there are limited experimental studies which examine the effect of SVA on normal and cancer cells of the human breast at the same time.

To examine any potential radiosensitizing or radioprotecting effect, an SVA concentration was needed which would not affect the MCF10A and MCF-7 cells on its own. By using cytotoxic analysis and growth curves, SVA concentrations up to 1 μM were identified for further studies. An SVA concentration of 3 μM did not affect the MCF-7 cells but caused a stagnation of MCF10A cell growth. Higher SVA concentrations, such as a concentration of 12.5 μM, caused an inhibition of cell growth in both cell lines (Supplement 2).

It could be assumed that the cytotoxic level of SVA differs between various cell types. For natural killer cells, an inhibition of the proliferation after 50 μM SVA could be observed [27]. Next to this, Crescencio et al. (2009) have analysed three cervical cancer cell lines and described strongly differed responses to SVA (10 μM up to 160 μM). The proliferation of CaSki- and ViBo cells was dose-dependently decreased, whereas HeLa cells showed an increase in the proliferation after 10 μM SVA, and afterwards, a dose-dependent decrease [28].

In further experiments, we investigated the effect of SVA on the radiation response of the two cell types. Except of the investigation of the clonogenic survival, an irradiation dose of 2 Gy as clinical relevant dose [29, 30] was used.

Early and residual γH2AX foci were measured to investigate the influence of SVA on the DNA repair capacity after IR. At an early time-point after IR, it indicates that with increasing concentration of SVA, the number of γH2AX foci decreased for MCF10A. But this effect was reversed for MCF-7—the number of γH2AX foci increased with increasing SVA concentrations. Determining the residual foci, a different pattern of the γH2AX foci number within the two cell lines was observed. In the MCF10A cells, increasing SVA concentrations caused a continuous increase in the mean number of DSBs for the irradiated cells. Without irradiation, the number of γH2AX foci was not affected in the MCF10A cells. In the MCF-7 cells, a concentration of 1 μM of SVA showed a significant increase in DSBs after the IR treatment. Lower doses of SVA caused no increase in the number of γH2AX foci. Interestingly, the unirradiated MCF-7 cells seemed sensitive for SVA—0.1 μM and 0.5 μM elicited a decrease in the number of γH2AX foci—whereas 1 μM of SVA slightly increased the number of γH2AX foci. So, SVA has an influence on the repair capacity of the cells, but this is not very clear: it is seen that 30 min after IR this effect is rather protective for MCF10A, whereas 24 h after irradiation it is rather sensitizing for MCF10A and MCF-7. Chen et al. (2018) have also observed an increase in the number of foci after a combination of SVA and radiotherapy (100 μM of SVA, 2 Gy) on prostate epithelial cells [31]. Interestingly, the number of foci was clearly affected in MCF-7 only by SVA at 24 h; the number increased for 1 μM and decreased for 0.1 μM and 0.5 μM.

This fact could be explained by the interaction between SVA and the key player in epigenetic regulation, as well as by further epigenetic changes like the downregulation of histone deacetylases—this effect of the SVA treatment was detected by Karlic et al. (2015) on different tumour cells [22]. Owing to this, it is possible that SVA could affect the number of γH2AX foci only by its epigenetic change capabilities.

After determining the influence of SVA on DNA repair capacity, the clonogenic survival was examined for further investigation concerning the long-term effects of a potential radiosensitization or radioprotecting effect of SVA. During the 2D cultivation, a radiosensitizing effect on MCF-7 and MCF10A was observable, especially at 6 Gy. For other cell types, such as gastric cancer, a synergistic effect of radiation and SVA (0.2 μM) was also observed [16]. In another study, four colorectal cancer cell lines also confirmed the radiosensitizing effect of SVA, a decreased number of colonies after the SVA treatment (1 μM and 2 μM), and a decreasing viability after a combined treatment with SVA and IR has been shown. [15]. Considering MCF10A as a representative of normal breast epithelial cells, it could be assumed that SVA had a small protective function. This could be attributed to the fact that at a concentration of 2.5 μM SVA, MCF10A cannot undergo a FGF2-driven malignant transformation in 3D cell cultures (led by using ultra-low attachment plates) [32].

It could be shown that the SVA-dependent radiation response varies within different breast cancer subtypes. MCF-7 cells are estrogen receptor (ER)^+^; progesterone receptor (PR)^+^, and Her2neu^−^ [33]. Epidemiological studies show that the receptor status of patients play an important role for the effectiveness of SVA. Because statin use is not associated with an improved overall survival of triple negative breast cancer patients [12], but a small subset of patients with ER^+^ tumours benefit from the use of statins [7]. It was also detected that MCF-7 cells, which are ER^+^, were not so strongly affected, like MDA-MB231 cells, as the representative for triple negative breast cancer [34]. But their experimental data is contrary to the epidemiological data from Shaitelman et al. (2017) [12] which showed no effect of statins in the cohort of triple negative breast cancer patients. So, it is conceivable that other breast cancer cell lines may respond differently to the SVA treatment depending on the severity of hormone receptors. But even the analysis of the combined effect of SVA and irradiation using a cell line with a different hormone receptor status, such as the triple negative breast cancer cell line MDA-MB231, showed no different results of clonogenic survival in comparison with the MCF-7 cells (ER^+^, PR^+^, Her2neu^−^; Supplement 3). Next to the hormonal status, the influence of inflammatory status of the breast cancer type could be important. If the effect on a particular cell type, such as mammosphere-initiating cells, within different types of breast cancer was investigated, a radioprotective effect of SVA on MCF-7, as a representative of non-inflammatory breast cancer, was shown in 2D and 3D cell cultures. Moreover, different cell lines of inflammatory breast cancer were radiosensitized by using SVA [35]. In contrast, the 3D culture was performed by using ultra-low attachment plates and not by a cell culture matrix like Matrigel™. Furthermore, the colony formation assays were only incubated for seven days, whereas 14 days of incubation were used in the present study. The different results of the present study and Lacerda et al. (2014) could possibly justified by focusing on different cell populations. As Lacerda et al. (2014) caused an enrichment of the cancer stem/progenitor cell population by using ultra-low attachment plates, basic fibroblast growth factor, epidermal growth factor, and B27 supplement. But no enrichment or selection of MCF10A cells was performed in the present study.

Moreover, it was interesting that the type of cultivation in 2D and 3D clearly affected the radiation response of MCF-7 at all doses after the SVA treatment. MCF-7 cells were much more radioresistant in 3D, while MCF10A cells were not affected as much. The radiation sensitivity of MCF10A in 2D and 3D did only differ at high irradiation doses. Several studies on mammary gland models have shown that the responses between 2D and 3D systems to irradiation can vary [36, 37]. Many differences could be found in breast cells when they are cultivated in 3D instead of 2D [38]. If mammary cells are cultivated in 2D, then crucial signals for metabolism, cell proliferation, differentiation, and cell death responsible for the formation of correct tissue-specific architecture and functions are lost [39, 40]. In addition to differences in the morphology and functional parameter of mammary cells, it has also been shown that, the tissue-specific gene expression and signalling pathways in 3D cultures are differently regulated than in cells cultivated in 2D cultures [39, 41]. In our study, the breast cancer cells were much more radioresistant in 3D—this has already been confirmed by Hehlgans et al. (2008) and substantiated with altered protein expressions and morphology [25]. The altered morphology and cellular association within the 3D culture may possibly influence the efficacy of SVA. For pancreatic carcinoma cells, it was already shown that the effectiveness of some drugs, such as gemcitabine and microtubule-inhibitors, decreased in 3D cultures compared to the 2D cultures [42]. Recently it was also shown that in pancreatic carcinoma the effect of statins were reduced in 3D compared to 2D cultures [43].

The influence of SVA on the cell cycle has been shown many times in the literature. After treatment with the drug, cell cycle arrests have been described which, depending on the cell type, occurred in the G0/G1 phase or the G2/M phase [44–46]. For example, a significant change in the cell cycle was found in MCF-7 cells due to a standstill in the G1 phase after SVA treatment [47]. The SVA concentrations used, at 2.5 μM and 40 μM, were significantly higher than in our studies. We observed that treatment with SVA at the nontoxic concentrations used generally did not affect the distribution of cells in the cell cycle phases of both cell lines, regardless of whether the cells were irradiated or not. Only after the higher concentration of 1 μM SVA an increase of MCF10A cells in G0G1 phase accompanied by a decrease of S phase cells could be detected. As already observed in the growth curves, the normal MCF10A cells were more sensitive than the MCF-7 tumour cells. During analyses of cell cycle distribution no Sub-G1 cells, an indication for apoptosis, could be observed. Cell death via apoptosis is characterized by DNA fragmentation. On the basis of their reduced DNA content, including nuclear condensation, which can be detected by flow cytometry as sub-G1 peak, apoptotic cells can be identified and quantified [48]. The results in our study revealed that as expected SVA treatment in the used concentrations may not induce apoptosis.

In recent studies it was shown, that SVA suppresses gene expression of survivin and the connective tissue growth factor (CTGF) in gastric and colorectal cancer cells [16]. These observations led those authors suppose that both, as radiation-sensitive genes known factors, may play an important role in SVA mediated enhancement of radiation sensitivity [16]. Based on the results of our study with breast cells we cannot confirm this conclusion. Although we observed a radiation-sensitizing effect in 2D in the colony formation assay, we could not determine any effect on the expression of survivin or CTGF when using the same SVA concentration of 1 μM. Other authors, however, reported an increase of SVA levels in tumour cells after irradiation with doses from 1 Gy to 8 Gy [49]. As already described, we did not see this effect in the used breast cell lines either.

As for the marker ERK1, its expression in the MCF-7 tumour cells was slightly increased by the radiation alone, but increased significantly after a combined treatment with SVA and radiation. It has been described in the literature that even low radiation doses of 0.05 Gy can induce activation of the ERK in MCF-7 cells [50]. We could also observe this effect for higher radiation doses (2 Gy). Although ERK1/2 has also been reported for MCF10A cells as a sensitive marker for the stress reaction after ionizing radiation [51], we could not observe an significant effect on the normal cells in our investigations.

## Conclusion

In our study, it has been proven that tumorigenic and non-tumorigenic human breast cells are affected by SVA alone or in combination with IR in terms of cell growth, clonogenic survival, and their DNA repair capacity. The results suggest that SVA may have, depending on cultivation conditions, a potential for radiosensitization. It is, therefore, important to further investigate the role of SVA in relation to the extent of radiosensitization and how it could be used to positively influence the therapy of breast cancer or other tumour entities. Future research should clarify the molecular mechanisms of how SVA affects the cellular survival.

## Supplementary Information

ESM 1 (PDF 505 kb)

## Data Availability

The data supporting this study are provided in the results section of this paper. Further datasets used and/or analyzed during the current study are available are stored by the authors at the University Medical Center Rostock.

## Abbreviations

2D: Two-dimensional
3D: Three-dimensional
CFA: Colony forming assay
CTGF: Connective tissue growth factor
DSB: Double strand break
ER: Estrogen receptor
ERK1: Extracellular-signal regulated kinase 1
HMG-CoA: 3-hydroxy-3-methylglutaryl coenzyme A
IR: Ionizing radiation
PR: Progesterone receptor
SF: Survival fraction
SF6: Survival fraction at 6 Gy
SVA: Simvastatin
