# Supplementary figures and images for: Simvastatin treatment varies the radiation response of human breast cells in 2D or 3D culture
Source: Invest New Drugs. 2020 Dec 11;39(3):658–69. doi: 10.1007/s10637-020-01046-6 (PMC8068713; doi:10.1007/s10637-020-01046-6)

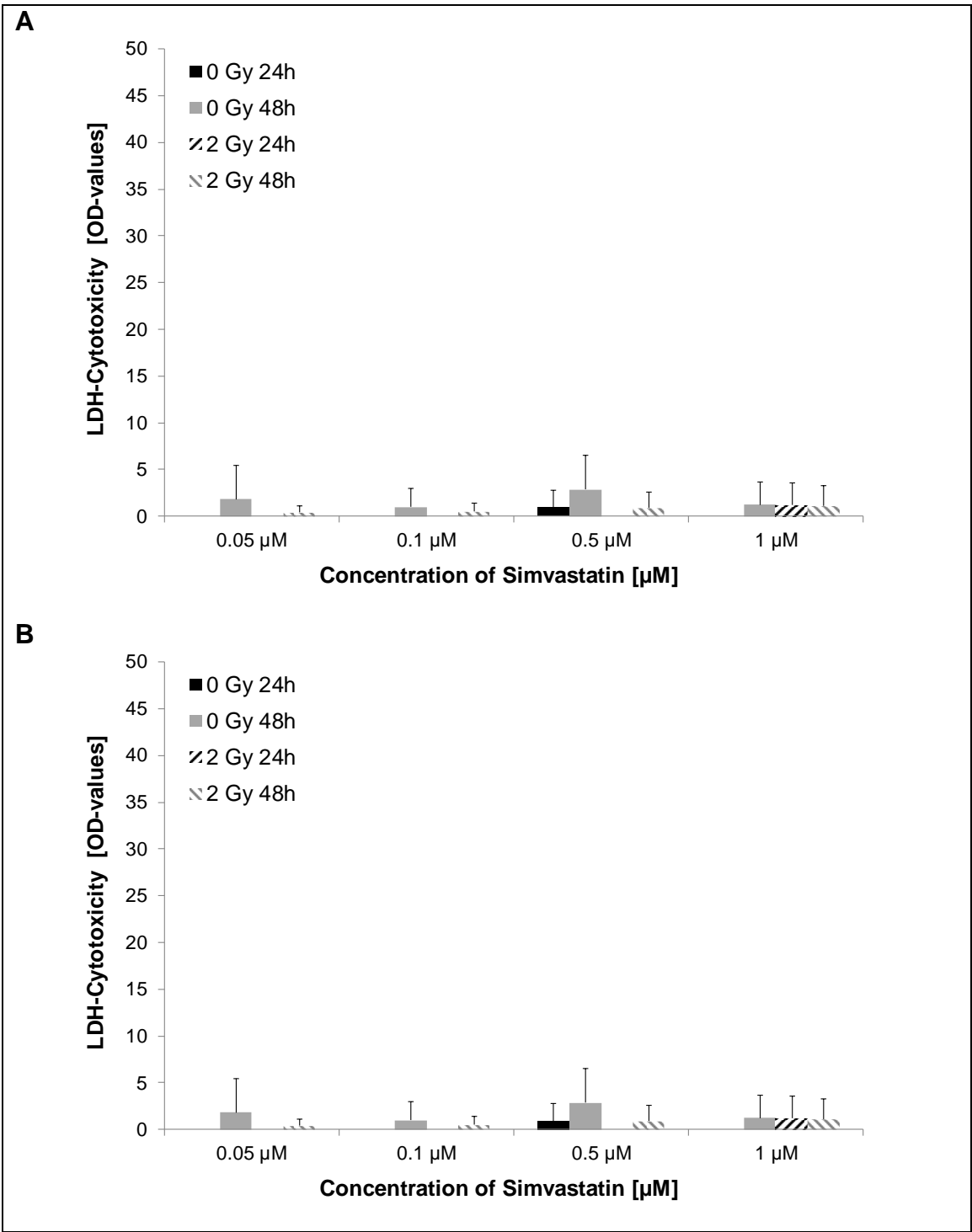

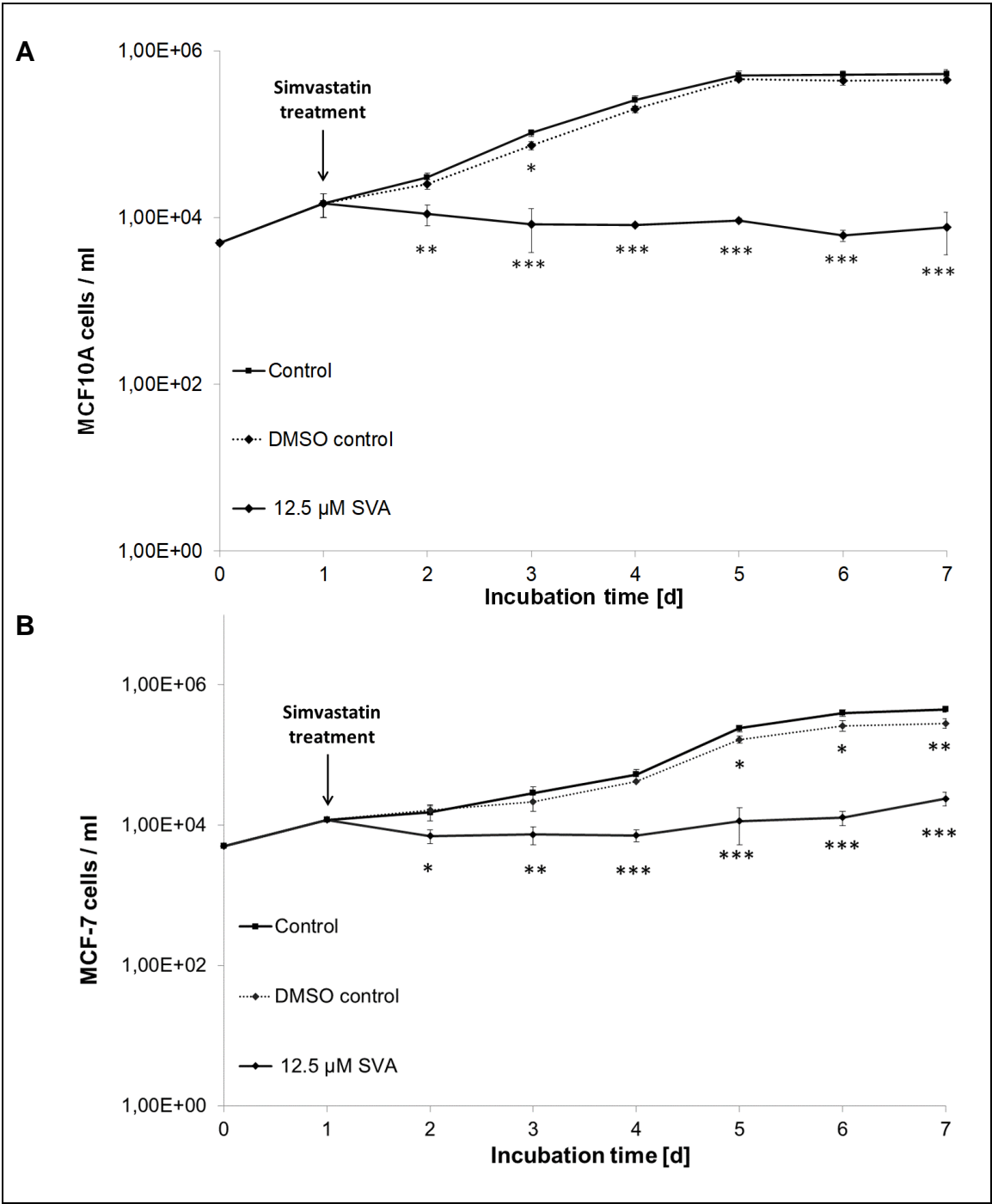

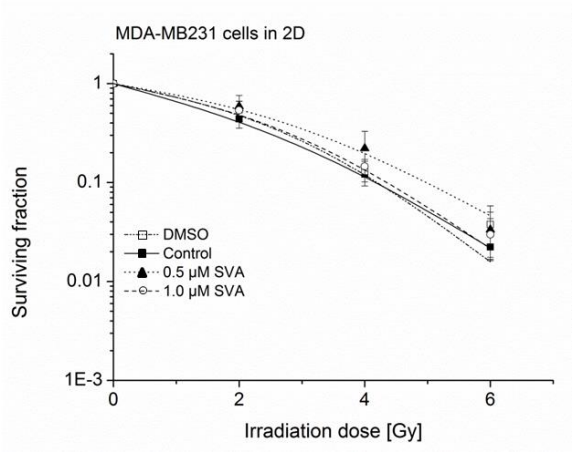

Supplement: Supplementary file 1 — (PDF 505 kb) [file 10637_2020_1046_MOESM1_ESM.pdf]
